# Application of action observation therapy in stroke rehabilitation: A systematic review

**DOI:** 10.1002/brb3.3157

**Published:** 2023-07-21

**Authors:** Chenping Zhang, Xiawen Li, Hongbiao Wang

**Affiliations:** ^1^ Department of Physical Education Shanghai University of Medicine & Health Sciences Shanghai China

**Keywords:** action observation therapy, stroke rehabilitation, systematic review

## Abstract

**Background:**

Numerous studies have described the positive effects of action observation therapy (AOT) on motor recovery among patients with stroke. However, there is no standardized procedure for when and how to intervene with AOT.

**Objectives:**

Thus, we reviewed and analyzed previous studies to provide a guideline for the application of AOT in stroke rehabilitation.

**Method:**

We searched PubMed, Cochrane Library, and EMBASE from inception to October 31 2022, using title and abstract search terms of “action observation” and “stroke” or “hemiplegia.” Of 4108 potential articles, 29 articles (sample size = 429 in AOT groups; sample size = 423 in control groups) that met inclusion criteria were included in final analyses.

**Results:**

The results suggested starting adjuvant AOT > 23 days after stroke onset and conducting 30–40 min/session, 3–5 times/week for at least 4 weeks.

**Conclusion:**

Based on our results, many factors will impact the effect of AOT on stroke rehabilitation, when to apply (timing) and how to apply (frequency, single, and total duration) should be fully considered when applying AOT as adjuvant therapy in stroke rehabilitation.

## Graphical abstract

The present review summarized the outcome measures employed in stroke patients and the scales and tests used to assess the outcome measures. When using adjuvant AOT with conventional therapy in stroke rehabilitation, we suggest starting AOT 23 or more days after stroke onset. Our results suggest 30–40 min per session may be most beneficial. We suggest that at least 4 weeks of treatment should be assessed in future studies. The review highlighted some tips for presenting AOT via video clips.

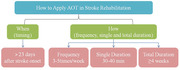

## 1 INTRODUCTION

The Global Burden of Diseases, Injuries, and Risk Factors Study (GBD) showed that in 2019, stroke was the second‐leading cause of death in the world and the third‐leading cause of death and disability combined (as measured by disability‐adjusted life‐years) (GBD 2019 Stroke Collaborators). More than 60% of stroke survivors experience motor deficits (Kwakkel et al., 2003) that seriously and negatively impact their quality of life. Several conventional physical therapies, such as constraint‐induced movement therapy, have been shown to have positive effects on the recovery from motor deficit among patients with stroke (Hakkennes & Keating, 2005). However, most of those conventional physical therapies require residual motor abilities to complete the physical practice, which may be difficult or even impossible for patients with stroke to perform. To overcome this limitation, some novel approaches, including action observation therapy (AOT) have been developed (Pomeroy et al., 2011). In a typical AOT procedure, participants watch video clips of people performing specific actions and then are asked to try to imitate the actions as best they can (see Buccino, 2014 for more details). The neural substrate by which AOT works may be related to the mirror neuron system (MNS), it was suggested that the MNS was activated both by the execution of action and by the perception of someone else performing the same action (Fadiga & Craighero, 2004; Gallese et al., 1996).

Numerous studies have described the positive effects of AOT for motor recovery among patients with stroke (Celnik et al., 2008; Franceschini et al., 2012; Sugg et al., 2015). Previous reviews indicated positive effect of AOT on the rehabilitation of stroke, some reviews using systematic review or meta‐analysis, suggested that AOT had a significant effectiveness on improving the upper limb motor functions, walking ability gait velocity, and daily activity performance in patients with stroke (Borges et al., 2018; Peng et al., 2019; Silverio et al., 2022; Zhang et al., 2019). Another review found that the benefit of incorporating AOT training into rehabilitation programs is strongly supported in populations with Parkinson disease and stroke (Ryan et al., 2021). However, there is no standardized procedure for when and how best to intervene during the treatment of patients with stroke. Thus, the aim of present study was to provide a guideline based on previous studies for the application of AOT in stroke rehabilitation.

## 2 MATERIAL AND METHODS

### 2.1 Search strategy

We conducted a literature search of PubMed, Cochrane Library, and EMBASE from inception to October 31, 2022, using the following search terms of titles and abstracts: “action observation” and “stroke” or “hemiplegia.” Articles in review articles were manually searched for potential inclusion.

### 2.2 Study selection

The studies selected included those with the following criteria: a longitudinal study published in English in a peer‐reviewed journal; participants with clinically diagnosed stroke; control group received sham AOT or no intervention; and behavior outcome measures were reported. Studies reporting only results of imaging or brain activity indexes were excluded.

### 2.3 Data extraction

The following data were extracted from the included studies for further analysis: (1) length of time from stroke onset; (2) sample size; (3) AOT protocol, including intervention duration for one session (e.g., 30 min/session), frequency (times/week), and intervention cycle (e.g., 4 weeks); (4) primary outcome measures; and (5) results of the intervention.

## 3 RESULTS

The literature search yielded 4108 potential articles. Following a review of titles and abstracts, 1459 duplicated articles and 711 registered trials without specific results were removed. Two authors then examined the remaining articles for adherence to the criteria defined in Section 2.2. A total of 29 studies were included in the final analyses.

### 3.1 Main characteristics of included studies

#### 3.1.1 Main characteristics of included studies

In the 29 included studies, there were 429 participants in AOT groups and 423 participants in control groups. In most studies (27 of 29, 93.1%), AOT was used as adjuvant therapy, with both AOT and control groups receiving conventional therapy. That is, the AOT group was treated with AOT in addition to conventional therapy, whereas the control group received only conventional therapy or only sham AOT intervention. Two studies (Lee et al., 2013; Oh et al., 2019) did not use conventional therapy during the experiments. Table 1 gives the main characteristics of the analyzed studies, including treatment regimens, behavior measures, and outcomes.

**TABLE 1:** Main characteristics of the 32 included studies.

Study	Stroke onset	Sample size		Treatment		AOT treatment details				Behavior measures	Results
		EG	CON	EG	CON	T/S	FRQ	DR (wk)	TD (min)		
Bang et al. (2013)	>6 months	15	15	AOT + conventional therapy	Conventional therapy	40	5	4	800	1. Balance ability: TUG; 2. Walking ability: 10‐m walk test and 6‐min walk test; 3. Gait function: KASW.	All measures: EG > CON
Cho and Kim (2020)	>6 months	15	15	Visual AOT with auditory stimulation + conventional therapy	Visual AOT + conventional therapy	30	3	8	720	Balance ability: static and dynamic.	EG > CON
Cowles et al. (2013)	3–31 days	9	13	AOT + conventional therapy	Conventional therapy	30*2	5	3	900	Arm function: Motricity; ARAT.	All measures: EG = CON
Ertelt et al. (2007)	>1 month	8	8	AOT + conventional therapy	Conventional therapy	90	5	4	1800	1. Motor function: WMFT; 2. Arm function: FAT; 3. Quality of life: SIS.	All measures: EG > CON
Fu et al. (2017)	2–6 months	28	25	AOT + conventional therapy	Conventional therapy	20	6	8	960	1. Motor function: FMA; WMFT; 2. Activities of daily living: MBI.	All measures: EG > CON
Franceschini et al. (2012)	EG: 31.0 ± 4.6 days CON: 29.5 ± 4.2 days (M ± SD)	48	42	AOT + conventional therapy	Conventional therapy	30	5	4	600	1. Motor function: FMA; MAS; 2. Arm function: FAT; BBT; 3. Activities of daily living: FIM.	BBT: EG > CON Others: EG = CON
Hioka et al. (2020)	16.6 ± 3.9 days (M ± SD)	8	8	AOT + conventional therapy	Conventional therapy	30	5	12	1800	1. Walking ability: FAC; 10‐m walk test; 2. Stroke scale: NIHSS; BRS; mRS; 3. Balance ability: TUG; 4. Motor function: NIHSS lower extremity.	10‐m walk test: EG > CON (but only for patients who could walk independently) Others: EG = CON
Hsieh et al. (2020)	1–6 months	7	7	AOT + conventional therapy	Conventional therapy	60	5	3	900	1. Motor function: FMA; 2. Arm function: BBT; 3. Activities of daily living: FIM; 4. Quality of life: SIS.	FIM: EG > CON Others: EG = CON
Kim et al. (2016)	1–6 months	11	11	AOT + conventional therapy	Conventional therapy	40	5	4	800	1. Motor function: FMA; MAS; 2. Arm function: BBT; 3. Activities of daily living: MBI.	MAS: EG = CON Others: EG > CON
Kim et al. (2016)	EG: 8.27 ± 1.98 months CON: 7.8 ± 1.78 months (*M* ± SD)	15	15	AOT + conventional therapy	Conventional therapy	30	5	4	600	1. Motor function: FMA; MAL; 2. Activities of daily living: MBI; 3. Wrist function: ROM.	All measures: EG > CON
Kim and Kim (2015)	>1 month	6	6	AOT + conventional therapy	Conventional therapy	30	5	6	900	Motor function: WMFT	EG > CON
Kim and Kim (2015)	>1 month	6	6	Purposeful AOT + conventional therapy	Conventional therapy	30	5	6	900	Arm function: Average velocity; Trajectory ratio; Motion angle	Average velocity & trajectory ratio: EG > CON Motion angle: EG = CON
Kim and Lee (2013)	>6 months	9	9	AOT + conventional therapy	Conventional therapy	30	5	4	600	1. Balance ability: TUG; FRT; 2. Walking ability: WAQ; FAC.	All measures: EG > CON
Lee et al. (2013)	>6 months	9	7	AOT	No therapy	10	5	3	150	Number of drinking motions	EG > CON
Lee et al. (2017)	>6 months	12	12	AOT + conventional therapy	only action observation without imitation + conventional therapy	30	3	6	540	1. Balance ability: BBS; 2. Walking ability: mEFAP.	All measures: EG > CON
Lee et al. (2020)	EG: 7.46 ± 1.61 months CON: 8.30 ± 1.97 months (*M* ± SD)	13	13	AOT + conventional therapy	Conventional therapy	30	5	4	600	1. Motor function: FMA; WMFT; MAL; 2. Activities of daily living: MBI.	All measures: EG > CON
Mancuso et al. (2021)	15.6 ± 8.3 days (*M* ± SD)	16	16	AOT + conventional therapy	Conventional therapy	30	5	4	600	1. Motor function: FMA; MAS; 2. Arm function: BBT; 3. Activities of daily living: FIM.	BBT & MAS: EG = CON FMA & FIM: EG > CON
Moon and Bae (2019)	≥12 months	7	7	AOT + conventional therapy	Conventional therapy	30	3	4	360	1. Walking ability: 10‐m walk test; 2. Balance ability: TUG; 3. Gait function: DGI.	All measures: EG > CON
Noh et al. (2019)	EG: 31.36 ± 16.87 days CON: 22.63 ± 7.00 days (*M* ± SD)	11	11	AOT + rTMS + conventional therapy	rTMS + conventional therapy	20	5	2	200	1. Motor function: FMA; 2. Arm and hand functions: MFT; grip power test.	All measures: EG = CON
Sugg et al. (2015)	>6 months	14	14	AOT + conventional therapy	Relaxation Sham + conventional therapy	60–90	7	2	840–1260	1. Motor function: FMA; MAL; 2. Arm and hand functions: FTHUE; CAHM.	All measures: EG > CON
Oh et al. (2019)	>6 months	17	18	Functional AOT	General AOT	30	5	4	600	Gait ability: assessed by GAITRite system; FGA.	All measures: EG > CON
Park et al. (2014)	>12 months	11	10	AOT + conventional therapy	Conventional therapy	30	3	4	360	1. Walking ability: 10‐m walk test; F8WT; 2. Gait function: DGI; gait symmetry.	All measures: EG > CON
Park et al. (2017)	>6 months	12	13	AOT + conventional therapy	Conventional therapy	30	3	4	360	1. Walking ability: 10‐m walk test; community walk test; 2. Balance ability: Activities‐specific Balance Confidence Scale; 3. Gait function: Spatiotemporal gait measures.	All measures: EG > CON
Park and Hwangbo (2015)	EG: 14.8 ± 6.1 months CON: 13.4 ± 8.2 months (*M* ± SD)	20	20	AOT + conventional therapy	Conventional therapy	30	5	8	1200	1. Balance ability: LOS; 2. Walking ability: TUG; 10‐m walk test.	TUG & 10‐m walk test: EG > CON Sway speed and total length of LOS: EG > CON Sway area of static balance: EG = CON
Sale et al. (2014)	30 ± 7 days (*M* ± SD) (range: 23–37 days)	33	34	AOT + conventional therapy	Conventional therapy	30	5	4	600	1. Motor function: FMA; 2. Arm function: BBT.	All measures: EG > CON
Shamsi et al. (2022)	>3 months	7	7	AOT + physical therapy	Physical therapy	52	3	4	624	Gait function: Spatiotemporal gait measures	Most gait measures: EG > CON
You et al. (2019)	1–6 months	21	21	AOT + conventional therapy	Conventional therapy	20	5	4	400	Language function: AOS; WAB; BDAE.	All measures: EG > CON
Yu and Park (2022)	>6 months	10	10	First‐person perspective AOT + conventional therapy	Third‐person perspective AOT + conventional therapy	30	5	4	600	1. Arm function: ARAT; MAL. 2. Activities of daily living: MBI.	All measures: EG > CON
Zhu et al. (2015)	EG: 30.67 ± 17.85 days CON: 31.54 ± 18.79 days (M ± SD)	31	30	AOT + conventional therapy	Conventional therapy	30	6	8	1440	1. Motor function: FMA; 2. Activities of daily living: MBI; 3. Arm function: MAS.	All measures: EG > CON

Abbreviations: AOS: apraxia of speech; AOT: action observation therapy; ARAT: Action Research Arm Test; BBS: Biodex Balance System SD; BBT: Box and Block test; BDAE: Boston Diagnostic Aphasia Examination; BRS: Brunnstrom recovery stage; CAHM: Confidence in Arm and Hand Movement Scale; CON: control group; DGI: Dynamic Gait Index; DR: duration; EG: experiment group; F8WT: Figure‐of‐8 walk test; FAC: Functional Ambulation Classification; FAT: Frenchay Arm Test; FGA: Functional Gait Assessment; FIM: Functional Independence Measure; FMA: Fugl‐Meyer Assessment; FRQ: frequency; FRT: Functional Reach Test; FTHUE: Functional Test of the Hemiparetic Upper Extremity; KASW: knee angle in swing phase during walking; *M*: mean; MAL: Motor Activity Log; MAS: Modified Ashworth Scale; MBI: Modified Barthel Index; mEFAP: Modified Emory Functional Ambulation Profile; MFT: Manual Function Test; mRS: modified Rankin Scale; NA: not available; NIHSS: National Institutes of Health Stroke Scale; ROM: wrist joint range of motion; SD: standard deviation; SIS: Stroke Impact Scale; T/S: No. of times/session; TD: Total duration; TUG: Timed Up and Go test; WAB: Western Aphasia Battery; WAQ: Walking Ability Questionnaire; WMFT: Wolf Motor Function Test.

#### 3.1.2 Functions evaluated and assessment measurements

The outcome measures included the ability to balance (8 studies, 27.6%), walking (8 studies, 27.6%), motor function (14 studies, 48.3%), arm and hand functions (11 studies, 37.9%), gait (6 studies, 20.7%), wrist function (1 study, 3.4%), language function (1 study, 3.4%), drinking motions (1 study, 3.4%), activities of daily living (8 studies, 27.6%), quality of life (2 studies, 6.9%), and stroke scale score (1 study, 3.4%). The scales and tests used to assess each outcome measure in the included studies are given in **Table** **2**.

**TABLE 2:** Scales and tests used to assess the indicated outcome measures in the included studies.

Measured function	Measuring tool
Balance	Activities‐specific Balance Confidence Scale (Filiatrault et al., 2007) Biodex Balance System SD (Biodex Inc., USA) Functional Reach Test (Duncan et al., 1990) Limits of Stability (Nichols, 1997) Timed Up and Go test (Podsiadlo & Richardson, 1991)
Walking ability	min walk test (O'Keeffe et al., 1998) 10‐m walk test (van Loo et al., 2004) Community walk test (Yang et al., 2008) Figure‐of‐8 walk test (Hess et al., 2010) Functional Ambulation Classification (Viosca et al., 2005) Walking Ability Questionnaire (Perry & Davids, 1992)
Gait function	Dynamic Gait Index (Jonsdottir & Cattaneo, 2007) Functional Gait Assessment (Wrisley et al., 2004) Gait function assessed by the GAITRite (CIR System Inc., Clifton, USA) Knee angle in the swing phase during walking measured using Dartfish motion analysis software (Dartfish, Switzerland) Modified Emory Functional Ambulation Profile (Wolf et al., 1999)
Arm and hand functions	Action Research Arm Test (Askim et al., 2010) Box and Block test (Mathiowetz et al., 1985) Confidence in Arm and Hand Movement scale (Chen et al., 2013) Frenchay Arm Test (DeSouza et al., 1980) Manual Function Test (Miyamoto et al., 2009) Motricity Index (Demeurisse et al., 1980) Functional Test of the Hemiparetic Upper Extremity (Wilson et al., 1984)
Global motor function	Fugl‐Meyer Assessment (Gladstone et al., 2002) Modified Ashworth Scale (Bohannon & Smith, 1987) Wolf Motor Function Test (Wolf et al., 1989)
Activities of daily living	Functional Independence Measure (Hamilton et al., 1987) Motor Activity Log (Uswatte et al., 2005) Modified Barthel Index (van Exel et al., 2004)
Stoke severity	Brunnstrom recovery stage (Brunnstrom, 1966) modified Rankin Scale (York, 2011) National Institutes of Health Stroke Scale (Lyden et al., 1994) Stroke Impact Scale (Duncan et al., 1999)
Others	Wrist joint range of motion measured with a goniometer (SG75, ± 2° error within a range of 90°, 10 Hz frequency, Biometrics Ltd, Newport, UK) Language function assessed by the Chinese apraxia of speech rating scale (Li, 2008) Western Aphasia Battery (Wang, 1997) Boston Diagnostic Aphasia Examination (Roth, 2011)

### 3.2 When to apply adjuvant AOT after stroke onset

Clinical guidelines have suggested implementing rehabilitation as soon as possible after stroke onset (National Stroke Foundation); however, this guideline may not work for adjuvant AOT with conventional therapy. Of 29 included studies, 22 (75.9%) included patients with stroke onset >1 month, and 7 studies (24.1%) included patients with stroke onset within 1 month. For patients starting intervention at the same time after stroke onset, those starting >1 month had better rehabilitation effects than patients who started intervention within 1 month. For studies with patients starting intervention at > 1 month of stroke onset, approximately 4 weeks’ AOT intervention provided more statistically significant changes in most outcome measures compared with the control group. By contrast, studies with patients starting intervention within 1 month of stroke onset found no or only a few significant differences in outcome measures compared with control group. Among the seven studies (Cowles et al., 2013; Franceschini et al., 2012; Hioka et al., 2020; Mancuso et al., 2021; Noh et al., 2019; Sale et al., 2014; Zhu et al., 2015) whose participants started intervention within 1 month of stroke onset, AOT had no or limited effects on stroke rehabilitation when the intervention duration was <8 weeks. For those patients, a much longer intervention duration was required to achieve significant differences between the AOT and control groups. Two studies (Cowles et al., 2013; Noh et al., 2019) did not find any difference between the AOT and control groups. Cowles et al. (2013) examined the effect of AOT (30 min/session, 2 times/day, 5 days/week, for 3 weeks; 900 min in total) among patients with stroke onset between 3 and 31 days (AOT group: 19.5 ± 7.0 days; control group: 17.8 ± 5.1 days). The outcome measures included arm function as measured by the Motricity Index (Demeurisse et al., 1980) and the Action Research Arm Test (Yozbatiran et al., 2008), and the results indicated no statistical significance between the AOT and control groups before vs. after treatment. Noh et al. (2019) also found no significant differences between the AOT and control groups after 2 weeks’ intervention (20 min/session, 5 days/week, 2 weeks; 200 min in total) among patients whose stroke onset was an average of 31.36 (± 16.87) days in the AOT group and 22.63 (± 7.00) days in the control group. Some studies found that only a few measured indexes showed significant differences between the AOT and control groups. For example, one study (Franceschini et al., 2012) examined global motor function as measured by the Fugl‐Meyer Assessment (Fugl‐Meyer et al., 1975) and the Modified Ashworth Scale (Bohannon & Smith, 1987), activities of daily living as measured by the Functional Independence Measure (Mathiowetz et al., 1985), motor and arm functions as measured by the Frenchay Arm Test and the Box and Block test among participants with similar intervention start times after stroke onset (AOT group: 31.0 ± 4.6 days; control group: 29.5 ± 4.2 days). That study found that only the Box and Block test showed significant differences in favor of the AOT group after 4 weeks of AOT (30 min/session, 5 days/week; 600 min in total). Similar results were found by Mancuso et al. (2021) (details in Table 1). Longer intervention durations may help to obtain significant results, but not in all cases. A study by Zhu et al. (2015) found that the scores for all the measured indices, which included the Fugl‐Meyer Assessment, Barthel Index, and Modified Ashworth Scale, were significantly better after 8 weeks’ intervention (30 min/session, 6 times/week; 1440 min in total) in the AOT group compared with controls (for stroke onset times; see Table 1). However, another study showed that for patients whose stroke onset was a mean of 16.6 ± 3.9 days, even when treatment was extended to 12 weeks (30 min/session, 5 days/week for 12 weeks; 1900 min in total), a significant improvement was observed in the AOT group compared with the control group only for the 10‐m walking test. In addition, this difference was found only for patients who could walk independently. No significant differences were found between the AOT and control groups for any of the other outcome measures assessed, including the National Institutes of Health Stroke Scale, Brunnstrom recovery stage, modified Rankin Scale, and Timed Up and Go test (Hioka et al., 2020). By contrast, when stroke onset was >23 days, the results were more favorable for AOT. For example, Sale et al. (2014) investigated the effect of AOT among patients whose stroke onset ranged from 23 to 37 days prior. They found that 4 weeks’ AOT intervention significantly increased patient motor function and arm function compared with standard rehabilitation alone.

Hence, when using adjuvant AOT with conventional therapy in stroke rehabilitation, we suggest starting AOT 23 or more days after stroke onset.

### 3.3 AOT intervention protocol

#### 3.3.1 Session duration

For the length of each intervention session, 30 min was used most often (18 of 29 studies, 62.1%), followed by 20 min (3 studies, 10.3%), 60 min (2 studies, 6.9%), 40 min (2 studies, 6.9%), 60–90 min (1 study, 3.4%), 52 min (1 study, 3.4%), and 10 min (1 study, 3.4%).

Considering that the time from stroke onset to the start of AOT may be a factor influencing the effect of AOT (see Section 3.2), we excluded seven studies with participants whose stroke onset was within 1 month of intervention. In the remaining studies, the length of each session varied from 10 to 90 min, with 30 min still being used most often (13 studies, 59.1%), followed by 40 min (2 studies, 9.1%), 20 min (2 studies, 9.1%), 60 min (1 studies, 4.5%), 60–90 min (1 study, 4.5%), 52 min (1 study, 4.5%), and 10 min (1 study, 4.5%).

As described in Section 3.2, for patients whose stroke onset was >1 month prior to starting AOT, patients in the AOT group had greater improvement in most outcome measures than patients in the control group. However, further analyses showed that in one study implementing AOT for 60 min per session (5 times/week, 3 weeks; 900 min in total), only Functional Independence Measure scores had a greater improvement in the AOT group than in the control group, with no significant differences between these groups for the Fugl‐Meyer Assessment, Box and Block test, and Stroke Impact Scale scores (Hsieh et al., 2020). Patients with stroke have difficulty maintaining concentration beyond 20 min (Simmons et al., 2008), which may explain why AOT for 60 min per session across 3 weeks did not result in beneficial effects. Therefore, when employing AOT in stroke patients, we should not only consider a minimum single session duration to ensure the efficacy, but also should be aware the single session duration not be too long which may beyond patients’ tolerance. Our results do not clearly indicate an optimal AOT session duration. However, combined with the characteristics of typical patients with stroke and the negative results of the study by Hsieh et al., our results suggest 30–40 min per session may be most beneficial.

#### 3.3.2 Frequency of sessions each week

Assessing all included studies, we found that most conducted AOT 5 times/week (20 studies, 69.0%), followed by 3 times/week (6 studies, 20.7%), 6 times/week (2 studies, 6.9%), and 7 times/week (1 study, 3.4%).

After excluding seven studies with stroke onset within 1 month of AOT, we found that most of the remaining studies also used 5 times/week (14 studies, 63.6%), followed by 3 times /week (6 studies, 27.3%), 6 times/week (1 studies, 3.4%), and 7 times/week (1 study, 3.4%).

Given that most studies conducted AOT either 5 times/week or 3 times/week, we screened for studies with the same amount of time per session and the same number of weeks of intervention. We found that although 5 times/week (30 min/session, 4 weeks) was most commonly used, some studies showed that AOT conducted 3 times/week (30 min/session, 4 weeks) also significantly improved walking ability, balance, and gait function in the AOT groups compared with the control groups (Moon & Bae, 2019; Park et al., 2014; Park et al., 2017).

#### 3.3.3 Overall intervention duration

Regarding the number of intervention cycles used, 4 weeks (16 studies, 55.2%) was the most common, followed by 8 weeks (4 studies, 13.8%), 6 weeks (3 studies, 10.3%), 3 weeks (3 studies, 10.3%), 2 weeks (2 studies, 6.9%), and 12 weeks (1 study, 3.4%).

After excluding seven studies in which patient stroke onset was within 1 month of the start of AOT intervention, 4 weeks (13 studies, 59.1%) remained the most common, followed by 6 weeks (3 studies, 13.6%), 8 weeks (3 studies, 13.6%), 3 weeks (3 studies, 13.6%), and 2 weeks (2 studies, 9.1%). Among those studies, most with treatments of ≥4 weeks reported positive effects of AOT compared with controls. Among the studies with treatment cycles < 4 weeks, a study by Sugg et al. (2015) using 2 weeks’ AOT (60–90 min/session, 7 times/week; 840–1260 min in total) demonstrated greater improvement in all measured indexes (Fugl‐Meyer Assessment, Motor Activity Log, Functional Test of the Hemiparetic Upper Extremity, and Confidence in Arm and Hand Movement Scale) compared with the control group. A study using 3 week's intervention (10 min/session, 5 times/week; 150 min in total) reported significant improvement in the number of drinking motions (Lee et al., 2013). However, another study using 3 weeks’ AOT (60 min/time, 5 times/week; 900 min in total) found significant differences between AOT and control groups only for Functional Independence Measure scores, but not for Fugl‐Meyer Assessment, Box and Block test, and Stroke Impact Scale scores (Hsieh et al., 2020).

Therefore, although additional corroborating evidence is required to conclusively determine the optimal number of weeks that patients with stroke should receive AOT, we suggest that at least 4 weeks of treatment should be assessed in future studies.

## 4 DISCUSSION

Most patients with stroke experience motor deficit (Kwakkel et al., 2003), some of them with severe motor deficit had difficult in receiving conventional therapy such as constraint‐induced movement therapy (Yavuzer et al., 2008). Thus, alternative therapies are needed to overcome this limitation. AOT is one of the novel approaches whose efficacy were approved by numerous studies (Buccino, 2014; Deconinck et al., 2015).

AOT was developed based on the neural substrate—the mirror neuron system (MNS), which was originally discovered in the monkey premotor and parietal cortex (Rizzolatti & Fogassi, 2014; Rizzolatti et al., 2014). Recent works suggested a comparable, distributed mirror neuron system in the human brain that is active during both action observation and execution. Neural activity associated with action observation includes visual processing areas (occipital, temporal, and parietal cortices) and the core mirror neuron system, which comprises motor‐related brain regions, including the pars opercularis of the inferior frontal gyrus (IFG), ventral premotor cortex (PMv), and rostral IPL (Grezes &Decety, 2001; Rizzolatti & Craighero, 2004). A large body of behavioral, neurophysiological, and brain imaging researched indicated that when we observe actions, our motor system simulates those action and increased corticospinal facilitation specific to muscles used to execute the observed actions was found (Urgesi et al., 2006). Moreover, Gazzola and Keysers (2009) assessed neural activity overlap during action observation and execution, and their results revealed relatively right‐left overlap in the temporal, parietal, and two frontal nodes, including strong activity in the mirror system. These findings provided us the neural perspective of how AOT work.

The present review investigated when/how to employ AOT as add‐on therapy, and our results suggested that adjuvant AOT should begin at least 23 days after stroke onset. The recommended adjuvant AOT protocol is 30–40 min/session, 3–5 times/week for at least 4 weeks. Also, we summarized some tips for presenting AOT via video clips. First, we would suggest the video to include auditory stimulation: A study by Cho and Kim (2020) found that the effect of AOT accompanied by rhythmic auditory stimulation was better than the effect of AOT with visual stimulation on the ability of patients with stroke to maintain their balance. Second, our results indicated that functional AOT is better than general AOT: Functional AOT differs from general AOT in that in the former, movements in real‐life situations are observed and then imitated and practiced. One study compared the effects of functional AOT and general AOT on gait function among patients with poststroke hemiparesis. In the functional AOT group, participants watched the daily activities video, which included clips of persons walking around the hospital, sitting and standing up from a toilet, shifting a food plate, and retrieving something from a refrigerator. In the general AOT group, participants watched a video that consisted of four clips of a person walking comfortably while (1) looking forward, (2) looking left and right, (3) looking up and down, and (4) looking forward, left and right, and up and down. Their results showed a greater degree of positive changes on gait function in the functional AOT group (Oh et al., 2019). Third, first‐person versus third‐person perspective should be considered: First‐person perspective action observation training was not only more effective in improving upper limb function and activities of daily living than third‐person perspective action observation training (Yu & Park, 2022) but also had higher localized and selective cerebral activation (Watanabe et al., 2011). The distinction between first‐person and third‐person perspective has been described elsewhere (Magill, 1998).

Besides, with the emerging of virtual reality‐based (VR) technology, VR rehabilitation treatments have been introduced to AOT. Several studies have approved the effectiveness of VR‐based rehabilitation in improving the upper limb motor function and daily life activity in stroke patients (Laver et al., 2017; Saposnik et al., 2010; Turolla et al., 2013). Hence, a combined rehabilitation treatment of AOT and VR‐based therapy would be recommended in the future.

There are several limitations that should be considered when interpreting the results of the present study. First, we only analyzed the behavior measures, the future studies should further analyze the brain activity measures to better verify the effectiveness of AOT. Second, we did not consider the impact of duration of the observed video, future study should take this factor into consideration. However, most of the included studies in present review considered the particular situation of stroke patients; they used short‐time videos (e.g. 3 min/video) and would ask the patients to take a rest between videos.

## 5 CONCLUSIONS

The aim of present systematic review was to offer a guideline on optimal times after stroke onset to begin adjuvant AOT and to provide intervention protocol suggestions as well as some tips on the contents of the AOT videos. Most studies used adjuvant AOT with conventional therapy for rehabilitation of patients with stroke. Our results suggested that adjuvant AOT should begin at least 23 days after stroke onset. The recommended adjuvant AOT protocol is 30–40 min/session, 3–5 times/week for at least 4 weeks. AOT video clips with rhythmic auditory stimulation provided better results than those with only visual stimulation. The use of videos with functional AOT rather than general AOT content and presented from the first‐person perspective rather than the third‐person perspective was associated with better results.

## CONFLICT OF INTEREST STATEMENT

The authors declare no conflicts of interest.

### PEER REVIEW

The peer review history for this article is available at https://publons.com/publon/10.1002/brb3.3157.

## Data Availability

The data that support the findings of this study are available from the corresponding author upon reasonable request.
